# Dietary Exposure to the Environmental Chemical, PFOS on the Diversity of Gut Microbiota, Associated With the Development of Metabolic Syndrome

**DOI:** 10.3389/fmicb.2018.02552

**Published:** 2018-10-24

**Authors:** Keng Po Lai, Alice Hoi-Man Ng, Hin Ting Wan, Aman Yi-Man Wong, Cherry Chi-Tim Leung, Rong Li, Chris Kong-Chu Wong

**Affiliations:** ^1^Department of Chemistry, City University of Hong Kong, Kowloon Tong, Hong Kong; ^2^Croucher Institute for Environmental Sciences, Department of Biology, Hong Kong Baptist University, Kowloon Tong, Hong Kong

**Keywords:** gut microbiome, bacterial diversity, microbiome-xenobiotic interaction, PFOs, energy metabolism

## Abstract

The gut microbiome is a dynamic ecosystem formed by thousands of diverse bacterial species. This bacterial diversity is acquired early in life and shaped over time by a combination of multiple factors, including dietary exposure to distinct nutrients and xenobiotics. Alterations of the gut microbiota composition and associated metabolic activities in the gut are linked to various immune and metabolic diseases. The microbiota could potentially interact with xenobiotics in the gut environment as a result of their board enzymatic capacities and thereby affect the bioavailability and toxicity of the xenobiotics in enterohepatic circulation. Consequently, microbiome-xenobiotic interactions might affect host health. Here, we aimed to investigate the effects of dietary perfluorooctane sulfonic acid (PFOS) exposure on gut microbiota in adult mice and examine the induced changes in animal metabolic functions. In mice exposed to dietary PFOS for 7 weeks, body PFOS and lipid contents were measured, and to elucidate the effects of PFOS exposure, the metabolic functions of the animals were assessed using oral glucose-tolerance test and intraperitoneal insulin-tolerance and pyruvate-tolerance tests; moreover, on Day 50, cecal bacterial DNA was isolated and subject to 16S rDNA sequencing. Our results demonstrated that PFOS exposure caused metabolic disturbances in the animals, particularly in lipid and glucose metabolism, but did not substantially affect the diversity of gut bacterial species. However, marked modulations were detected in the abundance of metabolism-associated bacteria belonging to the phyla Firmicutes, Bacteroidetes, Proteobacteria, and Cyanobacteria, including, at different taxonomic levels, *Turicibacteraceae, Turicibacterales, Turicibacter, Dehalobacteriaceae, Dehalobacterium, Allobaculum, Bacteroides acidifaciens, Alphaproteobacteria*, and *4Cod-2/YS2*. The results of PICRUSt analysis further indicated that PFOS exposure perturbed gut metabolism, inducing notable changes in the metabolism of amino acids (arginine, proline, lysine), methane, and a short-chain fatty acid (butanoate), all of which are metabolites widely recognized to be associated with inflammation and metabolic functions. Collectively, our study findings provide key information regarding the biological relevance of microbiome–xenobiotic interactions associated with the ecology of gut microbiota and animal energy metabolism.

## Introduction

The prevalence of non-communicable diseases (NCDs) is rapidly increasing, with cardiovascular diseases and diabetes being at the top of this list of NCDs, and multiple risk factors are widely recognized to be responsible for the increased incidences. Recently, the cumulative incidence of certain NCDs has been correlated to exposure to environmental chemicals (Gluckman et al., 2010). The past decades have witnessed the production of >150,000 synthetic chemicals, with approximately 2000 new chemicals being produced annually (Judson et al., 2009), and these heterogeneous chemical substances have been used for generating diverse industrial, agricultural, and commercial products. However, the release of these substances into the environment has adversely affected ecological and animal health. Depending on their chemical properties, these chemical substances have become dispersed in distinct environmental compartments and have contaminated food and water supplies. Retrospective analysis has revealed that exposure to various classes of environmental chemicals can occur through distinct routes and processes, including inhalation, dietary intake, and skin contact; this has resulted in the bodily accumulation of different environmental chemicals in the general population worldwide (Centers for Disease Control and Prevention, 2009), which indicates direct interactions of the exogenous chemicals within our body system.

The 2017 WHO global report on diabetes showed that >422 million adults were diagnosed with diabetes, underpinning the high prevalence of the disease and associated metabolic syndromes. People with a susceptible genetic background are predisposed to developing these diseases, and consumption of calorically dense diets and physical inactivity are the major risk factors associated with the disease development. However, these factors cannot account for the widespread prevalence of metabolic diseases in recent years, and thus additional investigation is required to reveal the pathogenesis of these diseases. Recently, scientific research has been focused on other potential risk factors that might disrupt body energy homeostasis, and considerable attention has been attracted by the roles of (1) gastrointestinal microbiota and (2) endocrine-disrupting chemicals (EDCs) as contributing factors.

The animal gut microbiome is a dynamic ecosystem formed by thousands of distinct bacterial species (Qin et al., 2010), and the remarkable metabolic activity in the gut environment is driven through a complex symbiotic interaction between these species. The gut bacterial diversity is shaped over time, with the complexity increasing due to the combined effect of multiple factors (such as genotype, diet composition, antibiotic therapy, and environmental exposure to xenobiotics) (Spor et al., 2011; Yatsunenko et al., 2012). The gut microbiota can potentially interact with environmental chemicals by altering the processes of absorption, disposition, metabolism, and excretion. Accordingly, gut bacteria have been widely reported to exhibit board ability to metabolize various environmental chemicals by using enzyme families (e.g., azoreductases, β-glucuronidases, β-lyases, nitroreductases, sulfatases) to catalyze diverse chemical reactions (e.g., reduction, hydrolysis, dehydroxylation, deacetylation, dinitration, deconjugation, demethylation) (Eriksson and Gustafsson, 1970; Williams et al., 1970; Bakke and Gustafsson, 1986; Rafii et al., 1990; Rafil et al., 1991; Roldan et al., 2008). A recent register of gut microbial biocatalytic reactions on xenobiotics listed 529 microorganisms that affect >1369 compounds (Gao et al., 2010); the study highlighted the capacity of gut microbes to transform diverse types of environmental chemicals. Notably, emerging evidence has indicated an association between body burden of environmental chemicals and gut microbial communities in the development of metabolic diseases (Alonso-Magdalena et al., 2011). In 2011, the U.S. National Toxicological Program studied the roles of environmental chemicals in the development of diabetes and obesity and reported positive correlations between EDC exposure and disease prevalence; the analysis prioritized the ten most predicted positive compounds across distinct biological processes: flusilazole, forchlorfenuron, d-cis/trans-allethrin, fentin, fludioxonil, niclosamide, prallethrin, thidiazuron, (Z,E)-fenpyroximate, and perfluorooctane sulfonic acid (PFOS). Among these chemicals, PFOS was listed as one of the risk factors for the development of metabolic diseases in the European research project OBELIX.

Alterations of gut microbiota composition are reported to be associated with various immune and metabolic diseases (e.g., inflammatory bowel disease, obesity, diabetes) (Cummings et al., 2003; Ley et al., 2006; Qin et al., 2012). However, few previous studies have investigated the interactions between environmental chemicals and gut microbiota and their toxicological relevance to the development of metabolic diseases. Here, we used a mouse model to assess the metabolic impact of dietary PFOS exposure. Physiological experiments and 16S rDNA metagenomic analyses were conducted to investigate the association among PFOS exposure, changes in gut bacterial community, and metabolic function.

## Materials and Methods

### Experimental Animals and Chemicals

Female CD-1 mice (6–8 weeks old), obtained from the Animal Unit of the University of Hong Kong, were housed in polypropylene cages containing sterilized bedding, maintained under a controlled temperature (23 ± 1°C, ambient temperature) and 12/12-h light/dark cycle, and provided *ad libitum* access to standard chow (LabDiet, 5001 Rodents Diet) and water (in glass bottles). The animal handling protocol was approved by the Committee on the Use of Human and Animal Subjects of the Hong Kong Baptist University (Permit no. 261812), in accordance with the Guidelines and Regulations of Department of Health, the Government of Hong Kong Special Administrative Region. The mice were acclimatized for 1 week before the PFOS-exposure experiments and then randomly divided into three groups (control, AC; low-dose PFOS, AL; high-dose PFOS, AH; at least four mice/group). PFOS (98% pure, Sigma-Aldrich) was dissolved in dimethyl sulfoxide (DMSO, Sigma-Aldrich) before mixing with corn oil; the final concentration of DMSO was <0.05% in all groups. The PFOS-exposure groups were weighed using an electronic balance (Shimadzu, Tokyo, Japan) and administered, every morning by oral gavage, 0.3 (AL) or 3 μg/g/day (AH) PFOS in corn oil for 7 weeks. The exposure doses were selected as described in our previous study (Lai et al., 2017), in reference with the human tolerable daily intake of PFOS established by the Scientific Panel on Contaminants in the Food Chain (European Food Safety Authority, 2008). The dose-range corresponded to the general population and occupational exposure levels. The control group (AC) received corn oil mixed with DMSO (0.05%).

Animals were sacrificed on Day 50 by cervical dislocation and cecal samples were collected. Blood samples were collected through cardiocentesis, and blood serum was prepared by centrifuging the samples at 3000 ×*g* for 15 min. The serum and the weighed liver samples were stored at -20°C and then used for triglyceride (TG) and PFOS measurements.

### Serum and Liver TGs

Serum and liver TG levels were quantified using the method described in our previous study (Wan et al., 2012) and a TG assay kit (Cayman, United States). Briefly, tissue samples were homogenized in chloroform:methanol (2:1) solution and then 0.05% sulfuric acid was added for phase separation. The aqueous phase was discarded, and the organic phase was collected and blow-dried under nitrogen gas at room temperature. The pellet was reconstituted in deionized water for 30 min at 37°C and then used for TG measurement.

### Chemicals and Instrumental Analysis for PFOS

We used a mass-labeled mixed standard solution for perfluorinated compounds (Product code: MPFAC-MXA; Lot number: MPFACMXA0714; >98% pure) from Wellington Laboratories (ON, Canada). Samples were extracted and analyzed as previously described (Wan et al., 2013). Briefly, each tissue sample was mixed with 2 ng of internal standard, 1 mL of 0.5 M tetrabutylammonium hydroxide solution, 2 mL of 0.25 M sodium carbonate buffer, and 5 mL of methyl tert-butyl ether, and this was followed by mixing in a reciprocating shaker (HS 501 digital shaker, Janke and Kunkel IKA Labortechnik) at 250 rpm for 20 min. The organic and aqueous layers were separated, and the organic phase was collected, and the extraction procedure was repeated and all organic phases were pooled. The solution was blow-dried under nitrogen gas (N_2_ ≥ 99.995%, Hong Kong Oxygen) in a nitrogen evaporator (N-EVAP112, Organomation Associates, Inc., MA, United States) and re-dissolved in 40% acetonitrile/60% 10 mM of ammonium acetate in Milli-Q water. An Agilent 1200 series liquid-chromatography system (Waldbronn, Germany) was used for PFOS detection. Chromatographic separation was performed using an Agilent ZORBAX Eclipse Plus C8 Narrow Bore guard column and an Agilent ZORBAX Eclipse Plus C8 Narrow Bore column. Tandem mass detection was conducted using an Agilent 6410B Triple Quadrupole mass spectrometer equipped with an Agilent Masshunter Workstation (version B.02.01) and an electrospray ionization source. The values of matrix recoveries were all 99%.

### Physiological Analysis

Oral glucose-tolerance test (OGTT) and intraperitoneal (i.p.) insulin-tolerance test (ITT) and pyruvate-tolerance test (PTT) were conducted on Day 50 on control and PFOS-exposed mice, as described in our previous study (Wan et al., 2014). Briefly, for OGTT, 16-h-fasted mice were administered glucose (2 mg/g body weight); for PTT, 16-h-fasted mice received an i.p. injection of sodium pyruvate (2 mg/g of body weight); and for ITT, 12-h-fasted mice received an i.p. injection of insulin (1 IU/kg body weight). For measuring blood glucose, blood samples were collected by means of tail prick at 0, 15, 30, 60, and 120 min. Area under the curve (AUC) values for OGTT, PTT, and ITT were calculated to evaluate glucose tolerance, the total glucose synthesized from pyruvate, and insulin sensitivity, respectively.

### 16S rDNA Metagenomic Sequencing

Cecal bacterial DNA was isolated using DNeasy Blood & Tissue Kit (Qiagen), according to manufacturer instructions, and 30 ng of qualified DNA was used to construct the library for metagenomic sequencing. V3-V4 Dual-index Fusion PCR Primer Cocktail and PCR Master Mix were used to amplify the V3-V4 regions of 16S rDNA, and the PCR product was purified using Ampure XP beads (Agencourt). The library was quantified using real-time quantitative PCR and was quality-checked using an Agilent 2100 bioanalyzer instrument (EvaGreen). The normalized library was subject to Illumina MiSeq sequencing for 250-bp paired-end sequencing; sequencing data have been deposited in the NCBI Sequence Read Archive (SRA)^1^, accession code SRP156864.

### Bioinformatics Analysis

To obtain accurate and reliable results in bioinformatics analyses, we used a dual-indexing approach (Fadrosh et al., 2014). Raw data were filtered to eliminate adapters and low-quality reads by using an in-house procedure; this included truncation of sequencing reads, based on the phred algorithm: the removed sequencing reads (1) were <75% of their original length and contained their paired reads; (2) included adapter sequences (default parameter: 15 bases overlapped by reads); (3) contained an ambiguous base (N base) and their paired reads; and (4) exhibited low complexity (default: reads containing the same base in 10 consecutive positions). For pooling the library with barcoded samples, the clean reads were assigned to corresponding samples by allowing 0 base mismatch to barcode sequences with in-house scripts. The data-processing results are listed in Supplementary Table S1. At least 2 Mbp of clean data were obtained from each sample, and the read-usage ratio was >70%. Paired-end reads featuring overlaps were merged to tags that were clustered to operational taxonomic units (OTUs) by using the scripts of USEARCH software (v7.0.1090) (Edgar, 2013). All tags were clustered to OTUs at 97% sequence similarity. Taxonomic ranks were assigned to OTU representative sequences by using Ribosomal Database Project (RDP) Naïve Bayesian Classifier v.2.2. Alpha-diversity analysis and the screening for different species were based on OTU and taxonomic ranks. Phylogenetic investigation of communities by reconstruction of unobserved states (PICRUSt) analysis was employed to predict functional capabilities by using our sequencing data (Langille et al., 2013).

### Statistical Analysis

Data are presented as means ± SEM. Differences between treatment and respective control groups were analyzed using Student’s *t*-test; *p* < 0.05 was considered significant. Analyses were conducted using SigmaStat for Windows.

## Results

### Effect of Chronic Dietary PFOS Exposure on Liver Weight and TG Content

**FIGURE 1:**
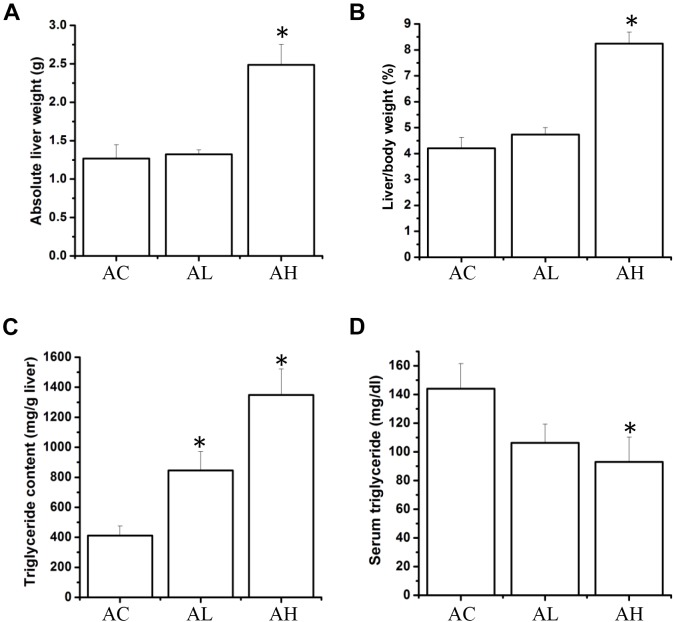
Effect of 7-week dietary PFOS exposure on liver weight and triglyceride content in mice. **(A)** Absolute liver weight, **(B)** liver index, **(C)** liver triglyceride level, and **(D)** serum triglyceride level were measured on Day 50 after PFOS treatment. Data are presented as means ± SD; ^∗^*p* < 0.05 versus control group. AC, control; AL, 0.3 μg/g body weight/day; AH, 3 μg/g body weight/day.

Upon completion of the PFOS-exposure study, on Day 50, the mean body weights were increased in the control (AC) group and the low-dose (AL) and high-dose (AH) PFOS-treatment groups, and the weights did not differ in a statistically significant manner among the groups. However, in the AH group, the liver was enlarged and the absolute liver weight was increased (Figure 1A), as was the ratio of liver weight to body weight (Figure 1B). The liver appeared yellowish in the AH-group mice (data not shown), which might be associated with lipid accumulation. Accordingly, measurement of liver TG content revealed a significant increase in the PFOS-exposed mice (Figure 1C), and the liver TG content was positively correlated with the increase in absolute liver weight. Intriguingly, serum TG content in the AH group was significantly decreased relative to control (Figure 1D). Table 1 shows the PFOS levels in both the liver and the serum in control and treatment groups; the accumulated PFOS levels were increased in a PFOS dose-dependent manner.

**Table 1:** Perfluorooctane sulfonic acid (PFOS) concentrations in liver and serum samples.

Sample name	PFOS (ng/g)	
	Liver	Serum
AC	7.10 ± 2.50	23.90 ± 8.18
AL	32942 ± 13473^∗^	33781 ± 4365^∗^
AH	503817 ± 325990^∗^	109526 ± 19371^∗^

*^∗^Statistically significant, *p* < 0.05 as compared with the AC group. AC-control, AL-0.3 μg/g body weight per day, and AH-3 μg/g body weight per day*.

### OGTT, ITT, and PTT

**FIGURE 2:**
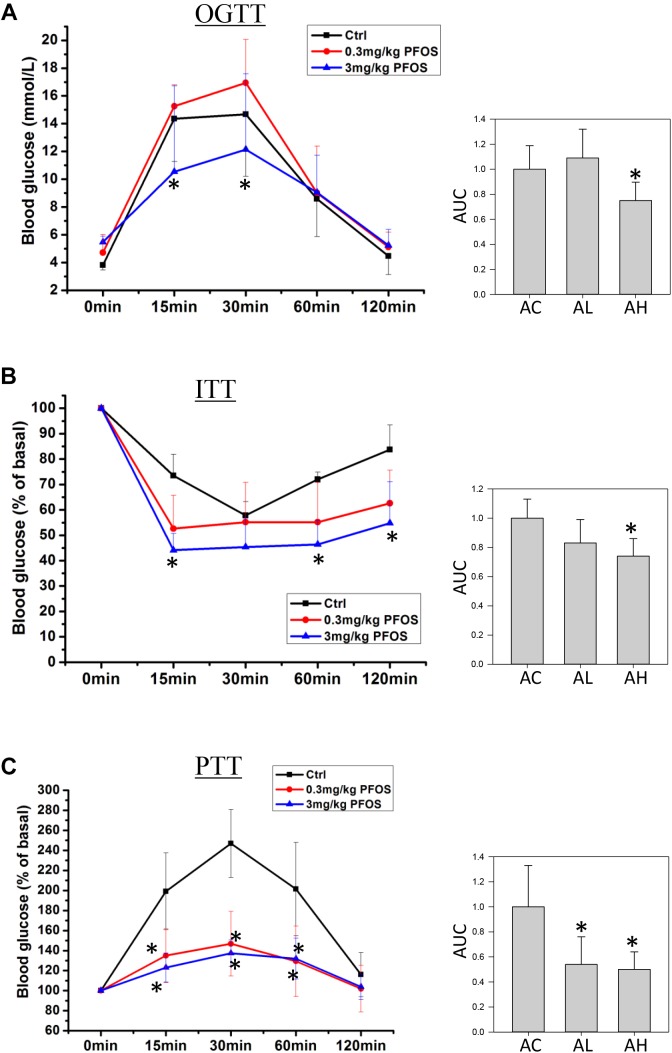
Effect of 7-week dietary PFOS exposure in mice, examined using **(A)** oral glucose-tolerance test (OGTT), **(B)** insulin-tolerance test (ITT), and **(C)** pyruvate-tolerance test (PTT). Left panels: changes in serum glucose levels against time in the assays; right panels: area under curve (AUC) values of the respective assays. Data are presented as means ± SD; ^∗^*p* < 0.05 versus control group. AC, control; AL, 0.3 μg/g body weight/day; AH, 3 μg/g body weight/day.

On Day 50, mice from the control and low- and high-dose PFOS-exposure groups were prepared for testing glucose metabolism and insulin function. OGTT results revealed that whereas the low-dose PFOS treatment did not significantly affect glucose tolerance (Figure 2A), the high-dose treatment elicited an earlier response in the reduction of blood glucose levels, at 15 and 30 min (*p* < 0.05), following glucose administration. The AUCs of OGTT were similar between the AC and AL groups, but the AUC of the AH group was significantly lower than that of the AC group (control).

Next, ITT-based measurement of body insulin sensitivity revealed that the mice in the AC and AL groups showed similar rate and extent of responses (Figure 2B), but in the AH-group mice, plasma glucose after insulin treatment was significantly lower than that in the control group. Accordingly, the AUC of the AH group was significantly lower than that of the AC group.

Lastly, to measure the effect of PFOS exposure on gluconeogenesis, pyruvate (a gluconeogenic substrate) was administrated and the rate of pyruvate conversion to glucose was measured. The PTT results indicated that pyruvate conversion in the AL and AH groups was significantly decreased relative to that in the AC group (Figure 2C).

### PFOS Exposure Exerted No Marked Effect on Gut Bacterial Species Diversity

**FIGURE 3:**
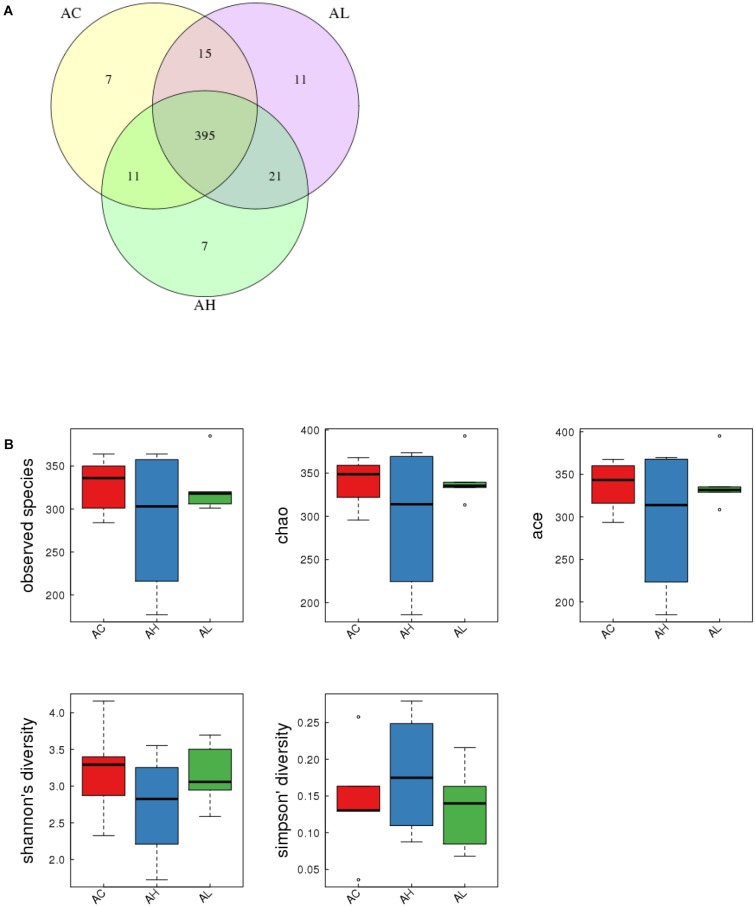
Effect of 7-week dietary PFOS exposure on gut bacterial structure in mice. **(A)** Comparison of operational taxonomic units (OTUs); different colors represent distinct groups: (i) control (AC), (ii) low-dose PFOS exposure (AL), and (iii) high-dose PFOS exposure (AH). The intersection represents the set of OTUs commonly present in the counterpart groups. Venn diagram was drawn using VennDiagram software R (v3.0.3). **(B)** Changes in observed species number, Chao1 index, Ace index, Shannon’s diversity, and Simpson’s diversity; the results suggest that dietary PFOS intake exerted no effect on the species diversity of the gut bacterial community.

To determine the changes in gut bacterial community caused by chronic dietary PFOS exposure, we performed metagenomic sequencing analysis on the V3-V4 regions of 16S rDNA; the DNA was collected from the ceca of mice in the AC, AL, and AH groups. In OTU analysis, we found that the degree of bacterial diversity was similar among the groups, and the predominant phyla included Firmicutes, Bacteroidetes, and Proteobacteria. In the sample AH4, the number of bacterial species was low (Table 2). Venn diagram analysis (Figure 3A) revealed that 395 OTUs were shared among the three groups. Alpha diversity was next applied for analyzing the complexity of species diversity in each sample by using several indices: observed species, Chao1, ACE, Shannon, and Simpson indices (Table 3). The rarefaction curves based on the observed species value and Chao1 and ACE data were used to evaluate the coverage of the sequencing. The result showed that the sequencing data were adequate for covering all the bacterial species in the community, which was reflected in the appearance of plateau regions in the curves from all the samples (Supplementary Figure S1). Moreover, comparison of the species diversity in the three groups revealed that the PFOS-exposure groups showed no significant differences in gut bacterial species diversity relative to the control group (Figure 3B).

**Table 2:** Operational taxonomic unit (OTU) analysis on each sample from the control (AC), low-dose (AL), and high-dose (AH) PFOS-exposed groups.

Sample name	Tag number	OTU number
AC1	67943	336
AC2	74582	301
AC3	74330	350
AC4	85158	284
AC5	88637	364
AH1	102682	351
AH2	91873	364
AH3	38690	255
AH4	75163	177
AL1	115877	385
AL2	55484	301
AL3	57234	318
AL4	57614	318
AL5	52056	306
AL6	71264	320

**Table 3:** Alpha diversity statistics in each samples from the control (AC), low-dose (AL), and high-dose (AH) PFOS-exposed groups.

Sample name	Observed species	Chao1	ACE	Shannon	Simpson
AC1	336	348.75	343.26	4.16	0.04
AC2	301	322.00	315.92	2.33	0.26
AC3	350	359.13	360.02	3.40	0.13
AC4	284	295.67	293.48	2.87	0.16
AC5	364	367.93	367.49	3.29	0.13
AH1	351	365.00	365.72	2.96	0.13
AH2	364	373.55	369.73	3.55	0.09
AH3	255	263.00	261.75	2.69	0.22
AH4	177	186.07	184.91	1.72	0.28
AL1	385	393.08	395.01	3.50	0.08
AL2	301	313.21	308.38	3.14	0.12
AL3	318	339.43	329.40	2.95	0.16
AL4	318	336.07	328.85	3.70	0.07
AL5	306	333.36	335.19	2.59	0.22
AL6	320	334.77	333.86	2.97	0.16

### PFOS Exposure Altered Gut Microbiome Community at Different Taxonomic Levels

**FIGURE 4:**
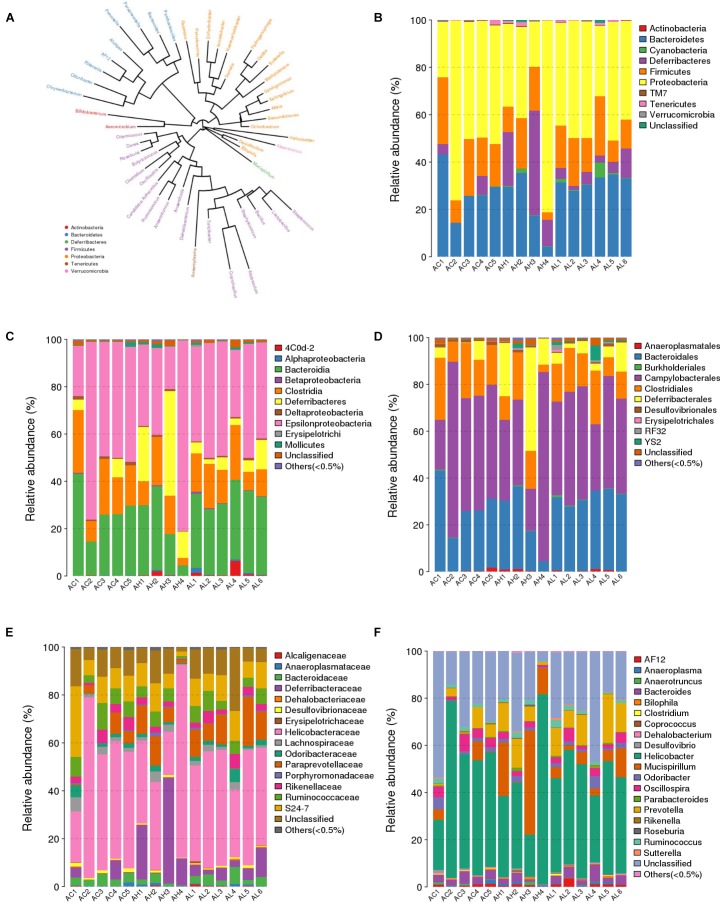
Effect of 7-week dietary PFOS exposure on gut microbiome community at distinct taxonomic levels. **(A)** Phylogenetic tree diagram at genus level. The same color indicates the same phylum. Taxonomic composition distributions in control (AC), low-dose PFOS-exposure (AL), and high-dose PFOS-exposure (AH) groups are shown at the levels of **(B)** phylum, **(C)** class, **(D)** order, **(E)** family, and **(F)** genus.

We compared the composition of the cecal microbiota at distinct taxonomic levels after dietary PFOS exposure. In the AL and AH groups, PFOS exposure produced similar and consistent effects in terms of changes in the abundance of certain microbial communities (Table 4). These changes included a significant increase at the level of the order *Turicibacterales* (belonging to the phylum Firmicutes) and a reduction of the species *Bacteroides acidifaciens* (phylum Bacteroidetes) (Figure 4A and Table 4); the increase in *Turicibacterales* was mainly contributed by an induction of the family *Turicibacteraceae* and genus *Turicibacter* (Figure 4A and Table 4). However, the abundance of certain other microbes was increased in either the AL group or the AH group: In the AL group, we identified a significant induction of the phylum Cyanobacteria (Figure 4B and Table 4), increases in *4Cod-2* (*Cyanobacteria-*like lineage) and the class *Alphaproteobacteria* (phylum Proteobacteria) (Figure 4C and Table 4), and an induction of the order YS2 (phylum Cyanobacteria) (Figure 4D and Table 4). Conversely, in the AH group, we detected a significant reduction in the family *Dehalobacteriaceae* (phylum Firmicutes) (Figure 4E and Table 4) and the genus *Dehalobacterium* (Figure 4F and Table 4). Collectively, our results demonstrated that dietary PFOS exposure led to changes in the abundance of specific members of the gut-microbiome bacterial community.

**Table 4:** Alteration of gut microbiome community at different taxonomy levels caused by dietary PFOS exposure.

Taxonomy level	Bacterial name	Ratio of low PFOS/normal diet	Ratio of high PFOS/normal diet
Phylum	*Cyanobacteria*	11.47*	4.98
Class	*Alphaproteobacteria*	19.25*	6.15
	*4C0d-2*	11.51*	5.01
Order	*Turicibacterales*	154.93*	68.49*
	*YS2*	11.51*	5.01
Family	*Turicibacteraceae*	154.93*	68.49*
	*Dehalobacteriaceae*	1.03	0.37*
Genus	*Dehalobacterium*	1.03	0.37*
	*Allobaculum*	9.82*	29.73
	*Turicibacter*	154.93*	68.49*
Species	*Bacteroides acidifaciens*	0.19*	0.23*

*^∗^Statistically significant change, *p* < 0.05*.

### PFOS Exposure Altered Gut Metabolism

Phylogenetic investigation of communities by reconstruction of unobserved states analysis was conducted to predict the functional profiling of gut bacterial communities in response to PFOS exposure. Our result demonstrated that both high- and low-dose PFOS exposure led to significant suppression of arginine and proline metabolism (Table 5). Moreover, high-dose PFOS exposure significantly reduced lysine biosynthesis and methane metabolism but induced butanoate metabolism. Taken together, these data suggest that PFOS exposure resulted in the alteration of gut metabolism.

**Table 5:** Alteration of gut metabolisms caused by dietary PFOS exposure.

Functional classification	Ratio of low PFOS/normal diet	Ratio of high PFOS/normal diet
Arginine and proline metabolism	0.92^∗^	0.93^∗^
Butanoate metabolism	1.04	1.06^∗^
Lysine biosynthesis	0.95	0.95^∗^
Methane metabolism	0.91	0.87^∗^

*^∗^Statistically significant change, *p* < 0.05*.

## Discussion

Perfluorooctane sulfonic acid represents a risk factor for the development of metabolic diseases. A cross-sectional study conducted using data from the U.S. National Health and Nutrition Examination Survey 1999–2000 and 2003–2004, which examined 474 adolescents and 969 adults, reported that high plasma concentrations of PFOS were associated with increased blood insulin levels (Lin et al., 2009). In an evaluation of a potential link between plasma PFOS levels in 571 Taiwanese workers and the risk of diabetes, elevated levels of the chemical were correlated with impaired glucose homeostasis and increased prevalence of diabetes (Su et al., 2016). Furthermore, experimental studies in animal and cell models have demonstrated that PFOS exposure alters glucose and/or lipid metabolism through perturbations of pancreatic β-cells, adipocytes, and liver function, and in studies on adult-stage animals, chronic PFOS exposure has been found to reduce body weight and fat, accompanied by an increase in liver mass (Lau et al., 2007; Martin et al., 2007; Zhang et al., 2008; Cui et al., 2009). In the previous studies, most experiments were conducted using high-dose and acute PFOS exposure, and the experimental setting was thus unlike that in the real-world scenario, where low-dose and chronic exposure occurs. Moreover, limited information on the roles of gut microbes in PFOS-exposed animals is currently available. Therefore, our study was designed to address this knowledge gap. In the biochemical analysis of body TG content, our data revealed hepatomegaly and lipid accumulation in the liver of AH-group mice. The observation of liver enlargement and lipid accumulation agreed with the results of our previous study in which we used higher PFOS doses (5 and 10 μg/g body weight/day) but a shorter exposure time (21 days) (Wan et al., 2012). The hepatic lipid content might be increased because of the impairment of lipid catabolism and/or hepatic lipid export; the reduction in lipid catabolism probably occurred due to an inhibition of β-oxidation, whereas the reduction in lipid transport was related to a downregulation of apolipoprotein B (Wan et al., 2012). This correlation was further supported by the results obtained in this study, which showed a marked reduction in serum TG level in the AH group. The perturbation of lipid metabolism could have affected glucose metabolism and insulin secretion (Antinozzi et al., 1998). Thus, we conducted physiological tests to evaluate the impact of PFOS exposure on glucose tolerance, insulin sensitivity, and hepatic gluconeogenesis. Our results showed statistically significant changes in the responses measured in OGTT and ITT in the AH group. The findings of both assays suggested that the high-dose PFOS exposure induced insulin hypersensitivity in mice, with the evidence indicating an increased rate of reduction of plasma glucose levels and a decreased rate of gluconeogenesis. The observation is supported by a previous study showing that exposure of mice to PFOA (perfluorooctanoic acid, a member of the PFOS family) led to an elevation of insulin sensitivity (Yan et al., 2015). One of the recognized physiological functions of insulin is to promote hepatic fatty acid synthesis. The high liver lipid content in the AH group appeared to be the biological outcome of this effect. Dietary PFOS exposure would lead to direct interaction of the chemical with the bacteria in the gut environment, and, intriguingly, this physiological outcome correlated with the changes in gut bacterial diversity assessed using 16S metagenomic analysis.

In our previous study in mice, we showed that daily intake of an environmental obesogen, bisphenol A, altered the gut bacterial structure (Lai et al., 2016). The pattern of the alteration was similar to that in high-fat-diet-fed mice. This observation supports the notion that environmental chemicals can perturb gut bacterial communities. In this study, we extended our investigation to address the effects of PFOS exposure on gut bacterial structure. Our results showed that chronic PFOS exposure (0.3 and 3 μg/g body weight, for 49 days) exerted no effect on gut bacterial diversity in general. However, when we examined specific taxonomic levels, we found that both low-dose and high-dose of PFOS exposure altered the abundances of distinct gut bacteria belonging to the phyla Firmicutes, Bacteroidetes, Proteobacteria, and Cyanobacteria. Some of these changes were reported to be associated with the symptoms of metabolic perturbations. For instance, PFOS exposure caused a marked induction of microbes in the order *Turicibacterales*, which was due to the growth of the bacteria in the family *Turicibacteraceae* and genus *Turicibacter*, and this induction was stronger in the low-dose PFOS-exposure group than in the high-dose group. A previous study showed that *Turicibacter* was increased in mice fed with a high-cholesterol diet, as compared with the level in the control group (Dimova et al., 2017); the data implied that *Turicibacter* was increased in response to the abundance of dietary cholesterol. Intriguingly, the results of an epidemiological analysis showed a positive correlation between serum PFOS and total cholesterol levels (Nelson et al., 2010). Moreover, other studies suggested that an increase in *Turicibacter* was correlated with dietary fat content, although the observations were inconclusive (Everard et al., 2014; Zhong et al., 2015). Nonetheless, the increase we observed here in the abundance of *Turicibacter* was likely related to the perturbing effects of PFOS on lipid metabolism. Another study on host–microbiota relationship in glucose-metabolism disorder demonstrated a positive association with *Turicibacteraceae* (Lippert et al., 2017). This association was observed here in our OGTT, ITT, and PTT data, particularly in the case of high-dose PFOS exposure. Moreover, following low-dose PFOS exposure, the abundance of the genus *Allobaculum* was increased substantially. *Allobaculum*, a putative short-chain fatty-acid-producing bacterium, was suggested to contribute to insulin resistance and obesity (Zhang et al., 2015). Besides this increase of bacterial abundance, our data revealed a marked reduction in the proportion of *B. acidifaciens* in the gut of mice in the PFOS-exposed groups, as compared with the proportion in the control group. *B. acidifaciens* is one of the predominant bacterial species responsible for promoting IgA production in the large intestine and is a specific commensal bacterium associated with amelioration of metabolic disorders in mice (Yanagibashi et al., 2013; Yang et al., 2017). The abundance of *B. acidifaciens* was found to be negatively correlated with liver TG levels in mice fed with a high-fat diet (Blasco-Baque et al., 2017), which supports our data indicating negative correlation between the levels of hepatic and serum TG in PFOS-exposed mice. The family *Dehalobacteriaceae* showed reduced abundance only in the high-dose group, which was contributed by the decrease in *Dehalobacterium*. In a study of 416 twin-pairs from the Twins population, a low abundance of *Dehalobacterium* was associated with a high body mass index and high blood lipid levels (Fu et al., 2015). The involvement of the gut microbiota in multiple metabolic pathways in the host is widely recognized, and, accordingly, the results of our PICRUSt analysis showed that PFOS exposure altered the microbial community functions, specifically in the metabolism of amino acids (arginine, proline, lysine), methane, and a short-chain fatty acid (butanoate). Alternations in the metabolism of these metabolites in intestinal bacteria were reported to affect host physiology (Dai et al., 2011; Nicholson et al., 2012); changes in arginine and proline metabolism were associated with coronary heart disease (Feng et al., 2016), whereas perturbations of butyrate and methane metabolism were related to inflammatory diseases (Morgan et al., 2012) and Type I diabetes (Brown et al., 2011). Furthermore, the GPR-43 receptor for short-chain fatty acids was demonstrated to be linked with fat accumulation in the host (Kimura et al., 2013). Retrospectively, we can conclude that our data on the changes in the abundance of gut bacteria and their metabolism in the PFOS-exposed groups were associated with the observed metabolic perturbations.

To our knowledge, this the first integrative study to report the effects of PFOS exposure on animal metabolism and gut bacterial community. Our data revealed that chronic PFOS exposure at 3 μg/g body weight/day induced insulin sensitivity, which was associated with an increase in hepatic lipid content but a reduction in hepatic gluconeogenesis. The results of intestinal 16S metagenomic analysis demonstrated marked changes in the abundances of bacteria at distinct taxonomic levels, including *Turicibacter, Allobaculum, B. acidifaciens*, and *Dehalobacteriaceae*; changes in the abundance of these bacteria are known to be associated with perturbations of glucose and lipid metabolism. Collectively, the results from this study implied that dietary PFOS exposure affected not only the glucose and lipid metabolism of the host animals, but also caused disturbance to the gut bacterial ecosystem. However, certain questions remain unresolved, such as the mechanistic interactions between PFOS and gut microbes and the changes in the production of bacterial metabolites, and further investigation in necessary to clarify the potential correlation between these changes and PFOS exposure.

## Data Availability

Sequence data generated in this study have been deposited in the NCBI Sequence Read Archive (SRA) (http://www.ncbi.nlm.nih.gov/sra); accession code: SRP156864.

## Author Contributions

KL participated in metagenomic sequencings, analyzed the data, and drafted the manuscript. HW, CL, and RL carried out the animal works and sample preparation. AW was involved in chemical analysis. AN carried out the bioinformatic data analysis. CW conceived the idea, formulated the hypothesis, and drafted the manuscript.

## Conflict of Interest Statement

The authors declare that the research was conducted in the absence of any commercial or financial relationships that could be construed as a potential conflict of interest.

## Footnotes

1 http://www.ncbi.nlm.nih.gov/sra

## Supplementary Material

FIGURE S1 Rarefaction curves based on Chao1 index, Ace index, and observed species values, showing that the data volume covered all species in the gut bacterial community.

TABLE S1 The sequencing statistic of metagenomics sequencing.
