# Supplementary figures and images for: Dietary Exposure to the Environmental Chemical, PFOS on the Diversity of Gut Microbiota, Associated With the Development of Metabolic Syndrome
Source: Front Microbiol. 2018 Oct 24;9:2552. doi: 10.3389/fmicb.2018.02552 (PMC6207688; doi:10.3389/fmicb.2018.02552)

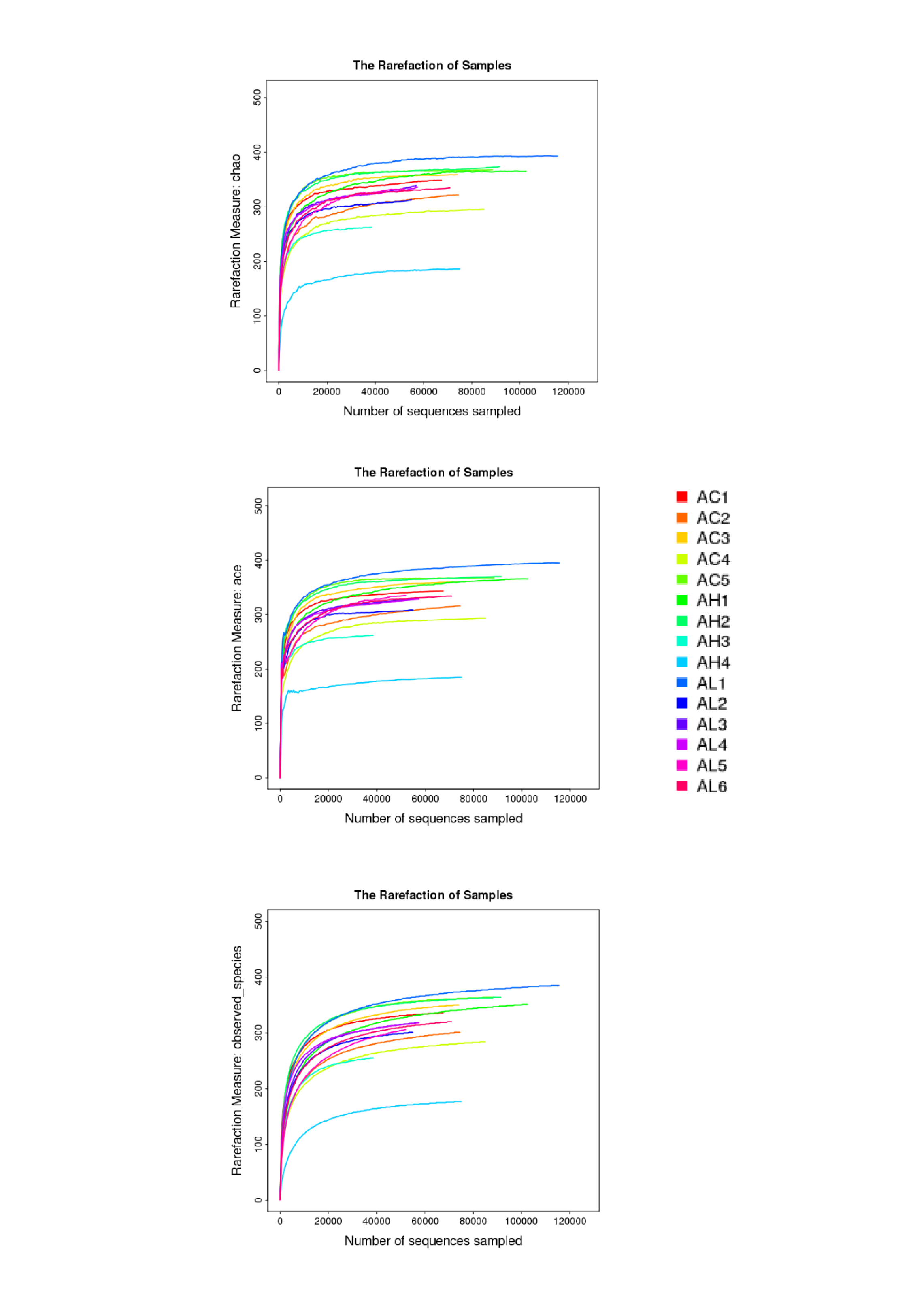

Supplement: FIGURE S1 — Rarefaction curves based on Chao1 index, Ace index, and observed species values, showing that the data volume covered all species in the gut bacterial community. [file Image_1.TIF]
