# Supplementary material for: Dietary Exposure to the Environmental Chemical, PFOS on the Diversity of Gut Microbiota, Associated With the Development of Metabolic Syndrome
Source: Front Microbiol. 2018 Oct 24;9:2552. doi: 10.3389/fmicb.2018.02552 (PMC6207688; doi:10.3389/fmicb.2018.02552)
Supplement: TABLE S1 — The sequencing statistic of metagenomics sequencing. [file Table_1.pdf]

SampleName	ReadsLength(bp)	RawData(Mbp)	Adapter(%)	NBase(%)	PloyBase(%)	LowQuality(%)	CleanData(Mbp)	DataUtilizationRatio(%)	RawReads	CleanReads	ReadUtilizationRatio(%)
**AC1**	297:294	57.4	0	0.001	0.003	14.568	38.58	67.2	97130*2	70159*2	72.23
**AC2**	296:294	55.17	0	0	0.002	9.955	41.9	75.95	93501*2	75694*2	80.96
**AC3**	294:294	63.3	0	0.002	0.002	14.11	43.22	68.29	107648*2	78882*2	73.28
**AC4**	293:294	72.32	0	0.003	0.008	13.355	50.51	69.85	123199*2	92307*2	74.93
**AC5**	300:294	70.33	0	0.001	0.003	11.885	50.91	72.39	118397*2	91536*2	77.31
**AH1**	300:293	88.89	0	0.002	0.031	13.54	61.44	69.12	149896*2	110819*2	73.93
**AH2**	299:293	79.22	0	0.003	0.003	14.378	53.49	67.53	133811*2	96562*2	72.16
**AH3**	298:293	33.37	0	0.004	0.064	15.277	21.75	65.19	56462*2	39568*2	70.08
**AH4**	296:293	54.8	0	0.001	0.019	9.977	41.8	76.28	93044*2	75528*2	81.17
**AL1**	299:294	103.55	0	0.003	0.013	12.596	73.41	70.89	174624*2	132337*2	75.78
**AL2**	298:294	45.18	0	0.005	0.005	13.189	31.33	69.34	76315*2	56706*2	74.31
**AL3**	297:293	45.71	0	0.002	0.009	13.234	31.99	69.98	77477*2	57936*2	74.78
**AL4**	296:293	48.64	0	0.002	0.004	14.815	32.46	66.73	82583*2	59029*2	71.48
**AL5**	294:293	42.55	0	0.003	0.014	14.285	29.03	68.23	72490*2	52849*2	72.91
**AL6**	293:293	63.1	0	0.001	0.018	15.865	41.27	65.4	107683*2	75701*2	70.3
